# FPGA-Based Multimodal Embedded Sensor System Integrating Low- and Mid-Level Vision

**DOI:** 10.3390/s110808164

**Published:** 2011-08-22

**Authors:** Guillermo Botella, José Antonio Martín H., Matilde Santos, Uwe Meyer-Baese

**Affiliations:** 1 Department of Computer Architectures and Automatic Control, Complutense University of Madrid, 28040 Madrid, Spain; E-Mails: jamartinh@fdi.ucm.es (J.A.M.H.); msantos@dacya.ucm.es (M.S.); 2 Department of Electrical and Computer Engineering, FAMU-FSU College of Engineering, Tallahassee, FL 32310, USA; E-Mail: umb@eng.fsu.edu

**Keywords:** bio-inspired systems, machine vision, optical flow, orthogonal variant moments, VLSI

## Abstract

Motion estimation is a low-level vision task that is especially relevant due to its wide range of applications in the real world. Many of the best motion estimation algorithms include some of the features that are found in mammalians, which would demand huge computational resources and therefore are not usually available in real-time. In this paper we present a novel bioinspired sensor based on the synergy between optical flow and orthogonal variant moments. The bioinspired sensor has been designed for Very Large Scale Integration (VLSI) using properties of the mammalian cortical motion pathway. This sensor combines low-level primitives (optical flow and image moments) in order to produce a mid-level vision abstraction layer. The results are described trough experiments showing the validity of the proposed system and an analysis of the computational resources and performance of the applied algorithms.

## 1. Introduction

There are several definitions of the goal of visual perception [1,2] as the interpretation of the information arriving at the retina, while a general agreement about the different abstraction levels and the limits between them is lacking.

Low-level vision obtains useful measurements such as colour, spatial frequency, binocular disparity, motion processing, *etc.*, from several channels. Some of these channels, or space-temporal filters, can be identified with receptive fields that deliver information to the retina. Others, such as binocular disparity or motion processing, are combinations of the previously mentioned ones.

Mid-level vision integrates primitives processed at a previous level. Information delivered at this stage corresponds to real-world inferences such as egomotion and independent moving objects (IMOs). They are called causal actions or object candidates in connection with any multimodal characterization. Examples of these are the combination of luminance measurements to infer lightness, shape from shading, perceptual grouping, figure organization, *etc.*

Finally, High-level vision interprets the scene through specific tasks such as relational reasoning, knowledge building, object recognition, *etc.* [1]

Regarding Low-level vision, optical flow considered as pixel motion estimation (velocity measure in terms of modulus and phase) of an image sequence, is an ill-posed problem due the inherent complexity of the signal processing tasks associated with it.

Motion processing has many important applications nowadays including robot navigation [3], biomedicine assistance [4], and so on [5]. Almost all complex computer vision systems include a core to specifically process motion, which will be then integrated with other early level primitives as mentioned above. These primitives are passed as input parameters to higher level vision stages. The applications mentioned here needs real-time capability when they are part of an embedded system, where the processing resources are constrained. There are some approaches [6] that only work with enough accuracy over a velocity range or noise free environment. Others suffer from contrast dependence or are unable to estimate second order motion [7,8].

On the other hand, moments in computer vision [9] are statistical measures which capture important information about an image, for instance, to describe its shape. Variant moments [10–13] are an alternative to the classic moment invariants. Variant moments are considered Low-Level processing because they process at the pixel level.

In this work, we present a prototype based on a FPGA device suitable for industrial applications which involves reduced size, rapid prototyping, low cost and power consumption. Our bioinspired sensor integrates two Low-level vision primitives represented by gradient family optical flow estimation and variant orthogonal moments. The optical flow platform provides the modulus and phase velocity values of each captured pixel. Orthogonal variant moments improve the robustness of the final system featuring the pixels. Both early-vision cues provide information for the Mid-Level output which has been configured in this contribution in the framework of segmentation and tracking tasks.

This paper is organized as follows, Section 2 provides a brief description of the different vision levels applied and the architecture of the whole integration. Section 3 describes the algorithms of the multimodal sensor. Section 4 presents the experimental results, the performance and the hardware resources needed. Section 5 summarizes the main innovative points, the comparison with other approaches and the presents the conclusions of the work.

## 2. Multimodal Platform

In this section the different Vision Levels applied are described. The final aim can be summarized in two challenges: the efficient integration of different primitives belonging to Low-level vision and the Mid-level vision processing module which gathers and computes data from the previously integration performed.

### 2.1. Pixel-Level Granularity: Low Level Vision

The starting point of the Low-level module of the platform is an improved FPGA-based implementation [14,15], which is briefly explained in this subsection. The optical flow Multichannel Gradient Model (McGM), designed by Johnston [16–20], was chosen to implement the Low-level vision system in VLSI due its robustness and bio-inspiration. This model deals efficiently with many challenges, such as illumination, static patterns, contrast invariance, robustness against failures, justification of some optical illusions [16], detection of second order motion and camouflage processing [16,17], *etc.* Its physical architecture and design principles are based on the biological nervous systems of mammalians [1,20–22]. At the same time, it avoids operations such as matrix inversion or iterative methods that are not biologically justified [16–18]. The original description of the McGM model [16–20] has been modified to improve the viability of the implementation in hardware.

Low-level vision processes the early visual information in a highly parallel and local way as the retina and primary visual cortex do [1,23]. The goal of this part is to estimate optical flow using a quotient of massively parallel bank of filters. These filters are obtained with a kernel function which depends on time and space. It conforms a bank filtering that progressively increases the order of the spatial (*r*) and temporal (*t*) differential operators involved in the kernel Equation (1):

(1) $$K(r,t)=\frac{1}{4\pi \sigma }e^{-\frac{r^{2}}{4\sigma }}\frac{1}{\sqrt{\pi }\tau \alpha \;\;\;e^{r^{2}/4}}e^{-{\left(\frac{\text{ln}(t/\alpha )}{\tau }\right)}^{2}}$$

where the parameters have been tuned to the follow values: *σ* = 1.5, *α* =10 and *τ* = 0.2. This expression is obtained following psychophysical and biological evidences from the mammalian and human visual systems [1]. It has been normalized and tuned assuming a human spatial frequency limit of 60 cycles/deg and a critical flicker fusion limit of 60 Hz [16].

After that, a tridimensional Taylor approximation of every pixel which depends on the derivative operators previously calculated from the kernel function is replaced by the intensity value. This expansion takes derivatives in time, *t*, and two spatial directions, *x* and *y*. These derivatives fit well with the receptive fields in the neural systems, there being multiple neurophysiological and psychophysical facts that support this processing scheme [1,16]. This system is biologically plausible and can be implemented by an artificial neural system in the visual cortex involving addition, multiplication and division of the linear spatial-temporal orientated filters [15]. The implemented model is a sequence of stages, where summarily their main concepts and associated task are described in the next paragraph:

Stage I accomplishes the temporal differentiation through fully stable and causal FIR filtering, convolving derivative operators of the kernel function (log-time domain Gaussian). It is important to notice that this implementation is different than that presented in previous works (IIR filtering) [14,15], achieving in this contribution longer delay although gaining in stability, modularity and scalability.

Stage II implements the spatial differentiation building functions of each temporal derivative previously implemented. This structure representation is computed via convolution with a set of neural “basis” filters modeled as derivatives of Gaussians.

Stage III steers each one of the space-time filters previously built at arbitrary orientations using a linear combination of other filters in a small “basis” set. Using the linear property of the convolution as main advantage, a filter *F_θ_* with orientation *θ* from the previous basic filter bank is formed. Many gradient optical flow models [2,7,8,24] can be implemented by just combining the outputs reached at this point.

Stage IV builds a Taylor expansion and its derivatives over *x*, *y* and *t* (denominated *X*,*Y*,*T* respectively) using the earlier calculated measures, delivering at the output a sextet which contains the products *XX*,*XY*,*XT*,*YY*,*YT*,*TT*. The Taylor approximation is truncated removing terms above first order in time and orthogonal direction accomplishing the fact of no more than three temporal filters and no greater spatial complexity in filters attending the biological proofs [25].

At this point, the whole information of the sequence of input frames is represented by a 3D structure where each pixel belonging to it can be reached in terms of a filter population tuned to different orientations and spatial frequencies.

Stage V forms four different functions called direct $\hat{s_{||}}$, $\hat{s_{⊥}}$, and inverse ${\overset{⌣}{s}}_{||}$, ${\overset{⌣}{s}}_{⊥}$ speeds where each pair of values is expressed using the plain and orthogonal components. These functions depend on the plethora of the different derivatives calculated before. The so-called *aperture problem* [24] inherent to optical flow is faced conditioning the raw values through a least square method applied to the different projections *θ*. These four functions are the velocity estimation primitives following the robustness and bioinspired nature of the model. The functions are combined, contributing either direct and inverse speed to the value accuracy due to the fact they are antagonistic and complementary enhancing strongly the robustness of the sensor. Additionally, there are several works supporting neurons which perform inverse speed measures [26,27], this fact also supplies an explanation of the sensitivity to static noise for motion blind patients [28].

Stage VI finally calculates two outputs: direction output from a measurement of phase that is combined across all speed related measures and the modulus output as a quotient of determinants, as shown in the following expressions:

(2) $$\mathrm{Modulu}s^{2}=\left|\frac{\begin{array}{cc} \hat{s_{||}}\text{cos}\theta & \hat{s_{||}}\text{sin}\theta \\ {\overset{⌣}{s}}_{⊥}\text{cos}\theta & {\overset{⌣}{s}}_{⊥}\text{sin}\theta \end{array}}{\begin{array}{cc} \hat{s_{||}}{\overset{⌣}{s}}_{||} & {\hat{s}}_{||}{\overset{⌣}{s}}_{⊥}\\ \hat{s_{⊥}}{\overset{⌣}{s}}_{||} & \hat{s_{⊥}}{\overset{⌣}{s}}_{⊥} \end{array}}\right|$$

(3) $$\mathrm{Phase}={\text{tan}}^{-1}(\frac{({\hat{s}}_{||}+{\overset{⌣}{s}}_{||})\text{sin}\theta +({\hat{s}}_{⊥}+{\overset{⌣}{s}}_{⊥})\text{cos}\theta }{({\hat{s}}_{||}+{\overset{⌣}{s}}_{||})\text{cos}\theta -({\hat{s}}_{||}+{\overset{⌣}{s}}_{||})\text{sin}\theta })$$

The complete optical flow Low-level vision model can be easily and gradually degraded to match previous models [18], even getting an ordinary optical flow Gradient model [7,8,29], as pointed out in a previous work [15].

### 2.2. Wave-Level Granularity: Low- and Mid-Level Vision

One of the most well established approaches in computer-vision and image analysis is the use of moment invariants. Moment invariants, surveyed extensively by Prokop and Reeves [9] and more recently by Flusser [11], were first introduced to the pattern recognition community by Hu [12,13], who employed the results of the theory of algebraic invariants and derived a set of seven moment invariants (the well-known Hu invariant set), which is now a classical reference in any work that makes use of moments. Since the introduction of the Hu invariant set, numerous works have been devoted to various improvements, generalizations and their application in different areas, e.g., various types of moments such as Zernike moments, pseudo-Zernike moments, rotational moments, and complex moments have been used to recognize image patterns in a number of applications [30].

The problem of the influence of discretization and noise on moment accuracy as object descriptors has been previously addressed by proposing several new techniques to increase the accuracy and efficiency of moment descriptors, deduction of the focus information from the second or fourth order central moments of a sequence of images [31], as well as methods for the efficient computation of certain classes of moments (e.g., Zernike moments, discrete orthogonal moments) [32–35]. Moreover, other works [36] address the same problem of Hu from different perspectives, e.g., achieving invariance to intensity, rotation, and scaling of color images based on the concept of principal component analysis and a competitive learning algorithm.

In short, moment invariants are measures of an image or signal that remain constant under some transformations, e.g., rotation, scaling, translation or illumination. Moments are applicable to different aspects of image processing, ranging from invariant pattern recognition and image encoding to pose estimation. Such moments can produce image descriptors invariant under rotation, scale, translation, orientation, *etc.* The general definition of moments of order *p + q* is as follows:

(4) $$M_{\mathrm{pq}}=\int \int x^{p}y^{q}f(x,y)dxdy\;\;\;\;\;\;\;\;\;\;\;\;;p,q=0,1,2,3,\ldots ,\infty$$

These moments produce a weighted description of *f*(*x,y*) over the entire image. The basis functions (*x^p^ y^q^*) may have a range of useful properties that may be passed onto the moments.

The method of variant moments [37] is a new technique for image analysis and computer vision that has many promising features for producing new kinds of very robust and simple computer vision algorithms. Variant moments possess a very simple definition; they are versatile and can be calculated very efficiently. They can also be used to characterize an image, object and scene for low, mid and high levels respectively. It seems very reasonable that one of its main areas of applications would be exploitation of the possible synergies with many other state of the art computer vision systems, e.g., optic flow-based techniques, as explained in this contribution.

Orthogonality means the decomposition an object, e.g., a point or vector, into, say, two components (its rectangular components *x*, *y*) in such a way that these two components are, *a priori*, uncorrelated, that is, it is possible to analyze how the object varies in one of its components, say *x*, in an independent way from the rest of the components, say *y.*

An *Orthogonal Variant Moment m* = *O(f)* is a measurement of a function *f* such that *m* varies if and only if the specific characteristic that is measured with this particular moment changes, that is, it is a measurement of an exclusive feature of a signal, image or wave form. Thus, an orthogonal variant moment set ***S*** is such that every element is uncorrelated with any other element of the set; in such a way that the value of some particular moment in an image sequence can vary while the remaining moments remain constant.

Invariants are sensitive to any image change or perturbation for which they are not invariant, so any unexpected perturbation will affect the measurements, that is, methods based on this approach can suffer from a high degree of uncertainty. On the contrary, a variant moment is designed to be sensitive to a specific perturbation, *i.e.*, to measure a transformation, not to be invariant to it and thus if the specific perturbation occurs it will be measured, hence any unexpected disturbance will not affect the objective of the measurement, that is, variant moments behave as specific detectors.

Assuming the restriction of two dimensional images on the plane, some useful orthogonal variant moments are the volume and area under the curve, the surface area ***S_a_*** computed by two orthogonal components (***L***_x_) for the *x*-axis and (***L***_y_) for the *y*-axis, an approximation of the phase of a wave which are called the position or station defined also in two orthogonal components ***P_x_*** and ***P_y_***.

Also, time derivatives of these orthogonal variant moments are used to obtain relevant measures about dynamic image sequences, for instance, measures of velocity and acceleration, ***V*** and ***A*** respectively, are obtained from the time derivatives of the position, *∂****P****_x_* and *∂****P****_y_*. The time derivatives of the surface area (length), *∂****L***_x_, *∂****L***_y_, represent the speed with which the disturbance is attenuated or amplified by a factor *k*. As long as the ratio between *∂L*_x_ and *∂L*_y_ remains constant, this fact can be interpreted as a zoom in/out from a perpendicular observer to the *xy*-plane.

The method introduced previously [37] operates by extracting, for each frame ***I*** of an image sequence or stream, a set ***M*** of moments, as shown in Equation (5):

(5) $$M(I)=[A(I);L_{x}(I);L_{y}(I);P_{x}(I);P_{y}(I)]$$

Once obtained the ***M*** vector, these moments can be used directly in several computer vision algorithms, for instance, to produce image segmentation, movement detection, shape analysis and object and pattern recognition.

### 2.3. Multimodal Sensor Architecture Integrated

**Figure 1.**
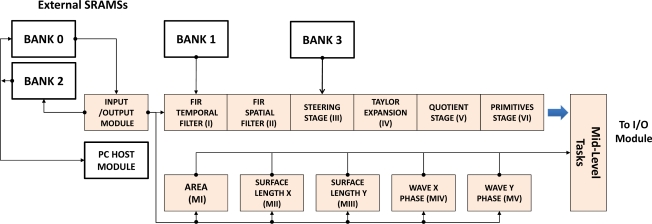
Scheme of the VLSI architecture of the Multi-Modal Sensor implemented in the FPGA.

The high level description tool Handel-C was chosen to implement this core within the DK environment [38]. The board used is the well-known AlphaData RC1000 [39] which includes a Virtex 2000E-BG560 chip and four SRAM banks of 2 Mbytes each. These external banks have been used for different implementations, accessing to them from both the FPGA and the PCI bus as shown in the Figure 1. Low-level optical flow vision is designed and built through an asynchronous pipeline where a message or token is passed to the next core each time one core finish the processing task. Nevertheless Low-level moment vision platform is implemented in a parallel way, being independent each one of the rest.

Each orthogonal variant moment and the optical flow scheme contribute to the final Mid-Level Vision estimation. The multimodal sensor core integrates the information from different abstraction layers (six modules for optical flow, five modules for the orthogonal moments and one module for the Mid-Level vision tasks). The Mid-Level vision core is arranged in this work for segmentation and tracking estimation with also an efficient implementation of clustering algorithm, although additional functionality to this last module can be added using this general architecture.

## 3. Algorithms of the Mid-Level Multimodal Sensor: Tracking & Segmentation Case Study

In this section the algorithms for performing tracking and segmentation are presented. Algorithm 1 (Segmentation function) shows a classical segmentation procedure that uses the well-known *k*-means clustering algorithm, although any other clustering algorithm could be used instead to group pixels into different classes. The *k*-means algorithm is implemented in hardware, thus modifying the structure proposed by [40], in order to reduce the computation time between the class centre and the pixels.

Every pixel is classified using a set of features for itself and a neighbourhood surrounding it, such as its *x,y*-coordinates, a set of orthogonal variant moments calculated for the subimage formed by the pixel’s neighbourhood *W_ij_* and additionally two components provided by the optic-flow subsystem indicating the magnitude *m_ij_* and the phase *θ_ij_*. Thus every pixel is represented by a vector of features *F_ij_* that will be classified into a cluster or class. The *k*-means algorithm has a quite critical parameter *k* which determines the number of different clusters to generate. One simple method to overcome this apparent limitation (due to the unknown possible number of moving objects in the scene) uses a large enough *k* and drops all insignificant or low quality clustering generated.

The full motion detection and tracking system is then achieved by the procedure described in Algorithm 2. The method is as follows: given an image sequence *S*, the algorithm will perform, for each temporal image frame, the segmentation procedure described above, in order to group the pixels of the current frame into different clusters. Once each valid cluster has been generated, every pixel will have a label indicating its class, e.g., 1, 2, 3, … *k*. With this starting information, the algorithms can proceed to superimpose a surrounding box over the image frame for each detected object. At this step, each cluster will represent a moving object and thus we can handle mid-level entities instead of low level entities (pixels).

**Algorithm 1.** The proposed integrated segmentation algorithm incorporating the variant moments and the measures of optic flow, flow’s magnitude and phase of each pixel (*m_ij_*, *θ_ij_*).

1:	**Function Segmentation(I)**
2:	{An image *I* of N × M pixel intensities}
3:	**for***i* = 1 to N **do**
4:	**for***j* = 1 to M **do**
5:	Obtain a window: $W_{\mathrm{ij}}=I\left[i-\frac{w}{2}\ldots i+\frac{w}{2},j-\frac{h}{2}\ldots j+\frac{h}{2}\right]\text{of}\;w\times h\;\text{neighbours of}\;I[i,j]$
6:	Obtain pixel features: $$F_{\mathrm{ij}}\leftarrow \left[\begin{array}{cc} \overset{x,y-\mathrm{coordinates}}{\overset{︷}{x(i),y(j),}} & \end{array}\begin{array}{cc} \underset{\mathrm{variant}-\mathrm{moments}}{\underset{︸}{A(W_{\mathrm{ij}}),L_{x}(W_{\mathrm{ij}}),L_{y}(W_{\mathrm{ij}}),P_{x}(W_{\mathrm{ij}}),P_{y}(W_{\mathrm{ij}}),}} & \overset{\mathrm{optic}-\mathrm{flow}}{\overset{︷}{m_{\mathrm{ij}},\theta_{\mathrm{ij}}}} \end{array}\right]$$
7:	**end for**
8:	**end for**
9:	*class-id* = *k-means*(*F,k,w*)
10:	**return***class-id*

**Algorithm 2.** The tracking algorithm used in the experiments.

**Require:** An image sequence *S.*	
1:	**for** each time step *t***do**
2:	*I_t_* ← new image frame from *S*
3:	class-id = **Segmentation**(*I_t_*)
4:	**for** each object in class-id **do**
5:	Update the object’s surrounding box based on pixel positions of class-id
6:	**end for**
7:	**end for**

## 4. Application to the Multimodal Bioinspired Sensor to Mid-Level Vision Tasks

In this section, the whole system is characterized according to the computational resources needed and the throughput obtained. Also, for the sake of clarity some visual results and a comparison with similar approaches are presented.

### 4.1. Computational Resources

Regarding the hardware resources, the metric for measuring the logic and the memory used will be the slice and the Block Ram occupation index. The software tool used to synthesize the final sensor under reconfigurable hardware (FPGA devices) is the ISE 12 suite [41].

The slower stage in the Low-level optical flow platform is Stage IV while Stage II needs the maximum number of Block RAMs due to the computations performed, as shown in Table 1. Stage V also needs a considerable amount of slices due the intensive use of multipliers. Some resources have been preserved in this implementation to be able to integrate all the optical flow and orthogonal moments in a whole system.

The number of cycles used (NC), determines the slower stage which restricts an improved throughput of the final system, regarding which, the Xilinx timing analyzer tool [41] delivers the results in terms of frequency around 25%–35% lower than the real frequency tested in our experiments. Table 1 also shows the performance of the optical flow scheme based on chained stages, attending to the pixel/seconds processed, is concluded that it is possible to compute real-time estimation with a resolution of 320 × 240 pixels.

Low-level orthogonal moments resources are presented in Table 2. Although the moments *L_x_* and *L_y_* represent the slowest part of the Orthogonal Moment scheme and they use more slices and Block Rams than *P_x_* and *P_y_*, in general, so these do not impose a resource limitation in the whole system.

Once each separate stage corresponding to an early-vision primitive is properly implemented, the integration and processing of the complete system is needed. Table 3 shows how the limits of the bioinspired global sensor are imposed by the Low-level vision platform, with the Mid-level vision acting as a supplement in terms of resources needed. In fact, the implemented platform has adapted the resources in comparison with previous works [14,15]; with this, the limit of the global system will be imposed by the slowest stage, awaiting the information from the asynchronous pipeline to be processed. Regarding that, it is important to remark that taking into account how the architecture has been designed the Mid-level task is one the last stages of the pipeline. The hardware requirements in term of slices, memory, number of cycles and performance for the implementation of the Multimodal Bioinspired Sensor can be seen in Table 3.

Table 4 finally shows the throughput obtained for several input resolutions of the global system expressed in Kpps (kilo pixels per second) and fps (frames per second). The maximum performance of the global system reaches up 2,000 Kpixels/second.

### 4.2. Visual Results

Three different experiments related to the processing of real input sequences captured from a static camera are displayed. The Low-level vision output indicates the optical flow estimation of each pixel using modulus and phase. On the one hand, the modulus (how fast the pixel is processed) is represented with a gradient intensity code, where black colour means no motion and white colour represents values with high velocity, on the other hand, the phase (direction towards which the processed pixel is moving) is represented using a colour coding as shown in the colour boundary frame. According to this formalism, downward motion will be represented using the blue tonalities, upward will use yellow tonalities and so on. Every pixel has individual information of its modulus and phase and every object has information about its segmentation and tracking surrounding area.

#### 4.2.1. Experiment I

**Figure 2.**
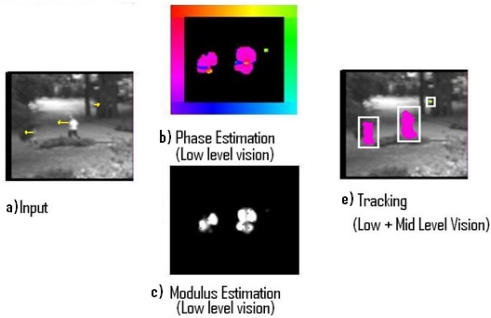
Results from Experiment I.

The first stimulus (Figure 2) represents two persons walking towards the left and showing a little residual motion in the central part of the frame sequence (a) with a resolution of 128 × 128 pixels. The motion is marked with yellow lines in order to indicate a qualitative approach. Phase Estimation indicates that the majority of the motion is moving towards the left (b). Modulus estimation gets a measure of the velocity of the pixels (c). Finally the tracking task follows the three different segmented objects (e).

#### 4.2.2. Experiment II

**Figure 3.**
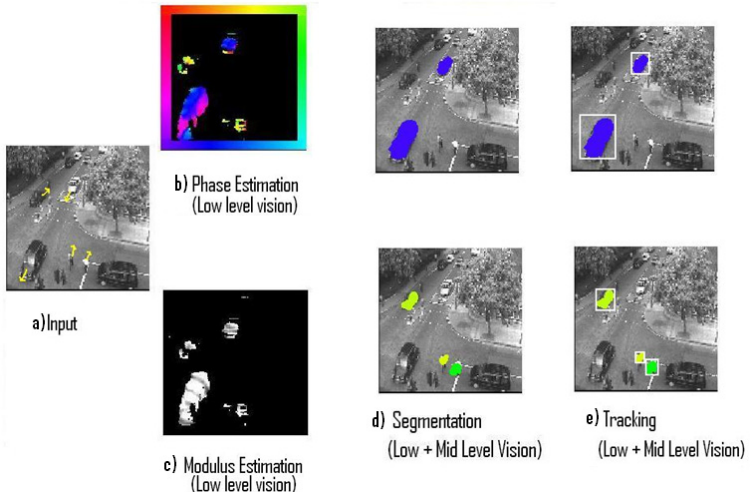
Results from Experiment II.

The second stimulus is a traffic sequence transition (Figure 3). There are different objects and speeds interacting (a) with a resolution of 128 × 128 pixels. Phase estimation delivers results moving towards down, right and up (b). Modulus estimation again provides velocity values (c). Segmentation (d) and Tracking (e) scheme processes five shapes.

#### 4.2.3. Experiment III

**Figure 4.**
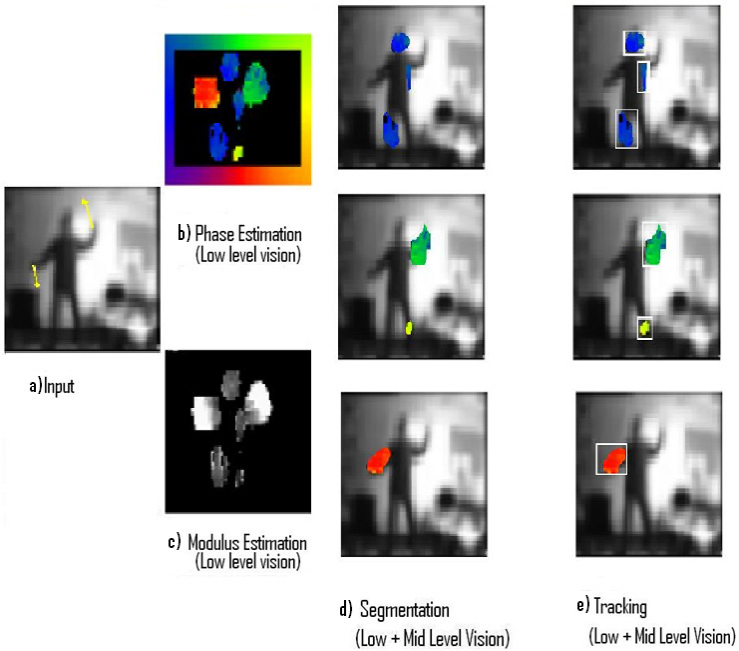
Results from Experiment III.

The third stimulus represents a person spreading their arms and legs upwards and downwards (a) with a resolution of 256 × 164 pixels (Figure 4). Phase estimation provides blue, green and red color values indicating motion towards the left, up and down. (b). Modulus estimation shows the different velocity values (c). Segmentation (d) and Tracking (e) process six contours.

### 4.3. Comparison with Other Approaches

Comparisons with other embedded complex vision models are presented in Table 5. The motion computation family and the method used are listed. The performance obtained and the computation densities are also shown. Every pixel value should be computed (100% density), nevertheless some of the methods below filter the inputs, reducing the processing space and thus the density.

There are many embedded engines regarding low-level vision [42–46]. This design reaches 2 Mpps, being able to deliver 26 frames/second with a resolution of 320 × 240 pixels, and a complete computation density (100%), thus enough for automation applications such as a little robot. It is important to remark that this model links two different abstraction layers, providing a Mid-level vision output.

Other approaches are based on motion estimation models (low-level) that are not biologically plausible; for example, the optical flow part of the presented model has been proved [16,17] to recover motion patterns based on texture-defined contours (second order motion) [47,48], which is very useful, e.g., for camouflage tasks and prediction the behaviour of many optical illusions.

## 5. Conclusions and Further Work

A complex bioinspired sensor, capable of computing multimodal low-level vision primitives to produce robust mid-level vision methods, is presented. The bioinspired sensor has been designed for Very Large Scale Integration (VLSI) using properties of the cortical motion pathway. This sensor combines low-level primitives (optical flow and image moments) in order to produce a mid-level vision abstraction layer. The whole system is scalable and modular, being it also possible to select the visual primitives involved (number of moments) as well as the bit-width of the filters and computation accuracy in the low-level vision (optical flow). This architecture can integrate different visual processing channels, so the proposed system makes possible the implementation of complex bioinspired algorithms on-chip.

In this respect, the integration of these low-level primitives through the proposed sensor has been applied to the design of a very efficient and robust visual tracking system. This specific system is robust in applications with high luminance variations and noisy environments. It is also useful in the research on the human perceptual system.

The integration of such different approaches represents a novel way of efficiently approaching complex computer vision systems. To the best of our knowledge, this is the first time that several low-level primitives are integrated with mid-level vision.

The integration of other low-level vision primitives such as phase, colour, motion, and binocular disparity is the next step in our research. It will also include mid-level inferences in the processing hence additional research will consider the combination of variant and invariant moments in the framework of low-level (pixel level) and mid-level (object level) vision and its integration with the optical flow. This complex vision system is currently being built on modern FPGAs using VHDL.

Furthermore, the computation of the multi-scale optical flow based on different moment measurements, instead of using the gradient based approaches of pixel intensity changes, and its hardware implementation, is a direct extension that is suggested by the presented model.

## Figures and Tables

**Table 1.** Slices, memory requirements, number of cycles and performance for the implementation of Low-level vision. Optical flow scheme.

**Low-level Vision Stage (Optical flow)**	**FIR Temporal Filtering I**	**FIR Spatial Filtering II**	**Steering III**	**Product & Taylor IV**	**Quotient V**	**Primitives VI**
Slices (%)	190 (1%)	1307 (7%)	1206 (6%)	3139 (19%)	3646 (20%)	2354 (12%)
Block RAM (%)	1%	31%	2%	13%	16%	19%
MC	13	17	19	23	21	19
Throughput (Kpixels/s)/Frequency limited by ISE tool (MHz)	4,846/63	3,235/55	2,526/48	1,782/41	1,695/39	2,000/38

**Table 2.** Slices, memory requirements, number of cycles and performance for the implementation of Low-level vision. Orthogonal moment scheme.

**Low-level Vision Stage (Orthogonal Variant Moments)**	**Area (M_I_)**	***L_X_* (M_II_)**	***L_Y_* (M_III_)**	***P_X_* (M_IV_)**	***P_Y_* (M_V_)**
Slices (%)	321 (2%)	1245 (7%)	1245 (7%)	658 (4%)	658 (4%)
Block RAM (%)	1%	4%	4%	3%	3%
MC	7	11	11	5	5
Throughput (Kpixels/s)/Frequency limited by ISE tool (MHz)	4546/49				

**Table 3.** Slices, memory requirements, number of cycles and performance for the implementation of Low and Mid-Level vision. Multimodal Bioinspired Sensor.

**COMPLETE Mid-level and Low level Vision**	**Motion Estimation (Low-Level)**	**Orthogonal Variant Moments (Low-Level)l**	**Tracking & Segmentation Unit (Mid-Level)**	**Multimodal Bioinspired Sensor. (Mid-level & Low-Level)**
Slices (%)	4127 (24%)	11842 (65%)	1304 (6%)	17710 (97%)
Block RAM (%)	15%	80%	4%	(99%)
MC (limiting)	29	11	18	29
Throughput (Kpixels/s)/Frequency limited by ISE tool (MHz)	4546/49	2000/38	2000/38	2000/38

**Table 4.** Throughput in terms of Kpps and frames/second for the embedded sensor.

**COMPLETE Mid-level and Low-level Vision**	**Orthogonal Variant Moments (Low-Level)l**	**Motion Estimation (Low-Level)**	**Multimodal Bioinspired Sensor. (Mid-level & Low-Level)**
resolution 120 × 96	395 frames/s	174 frames/s	174 frames/s
resolution 320 × 240	59 frames/s	26 frames/s	26 frames/s
resolution 640 × 480	28 frames/s	14 frames/s	14 frames/s
Throughput	4546 Kpixels/s	2000 Kpixels/s	2000 Kpixels/s

**Table 5.** Comparison with other complex system vision approaches.

**Models**	**Family**	**Method**	**Throughput (Mpixel/s)**	**Density**
Present work	Gradient	Enhanced McGM and Orthogonal variant moments	2	100%
Botella *et al.* [14,15] (2009, 2010)	Gradient	McGM	0.2	100%
Wei *et al.* [42] (2008)	Gradient	Horn & Schunck	4	100%
Diaz *et al.* [43] (2007)	Gradient	Lucas & Kanade	82	57.2%
Tomasi *et al.* [44] (2010)	Energy	Phase Based	49	not provided
Sosa *et al.* [45] (2006)	Gradient	Horn & Schunck	1.8	not provided
Mahalingam *et al.* [46] (2010)	Gradient	Lucas & Kanade	9.9	6.3%
